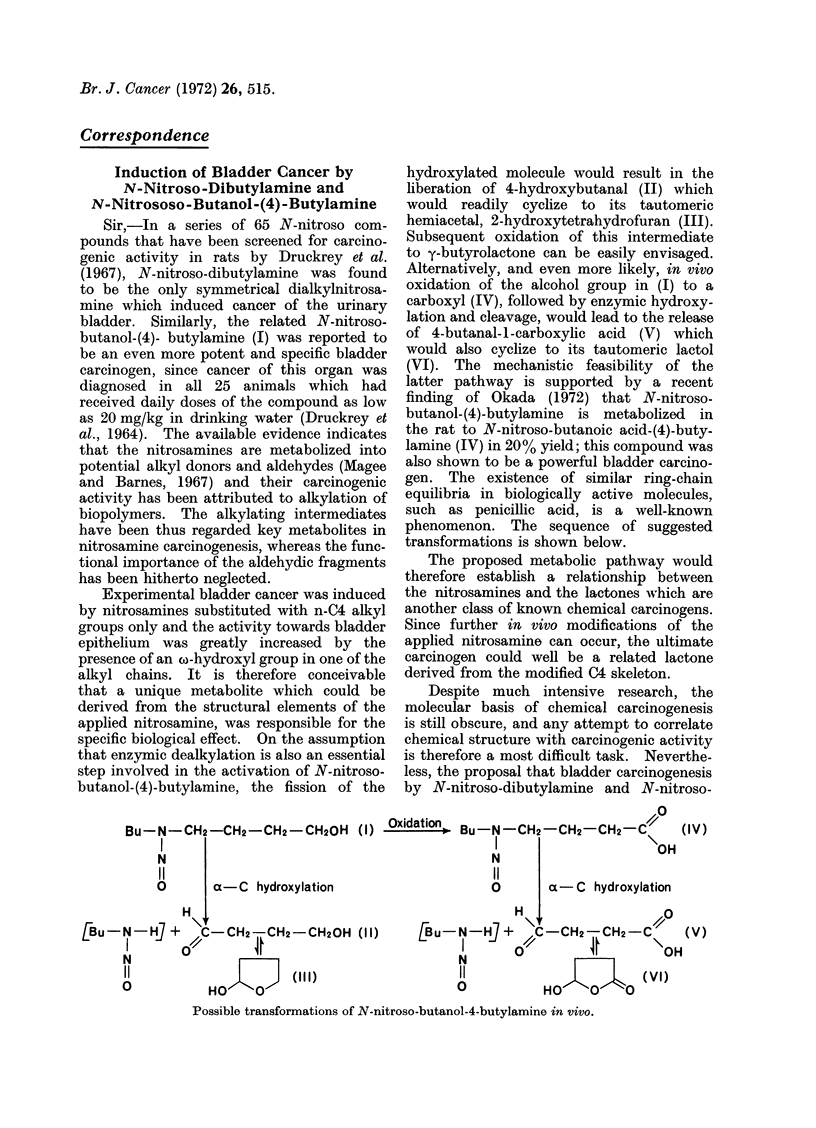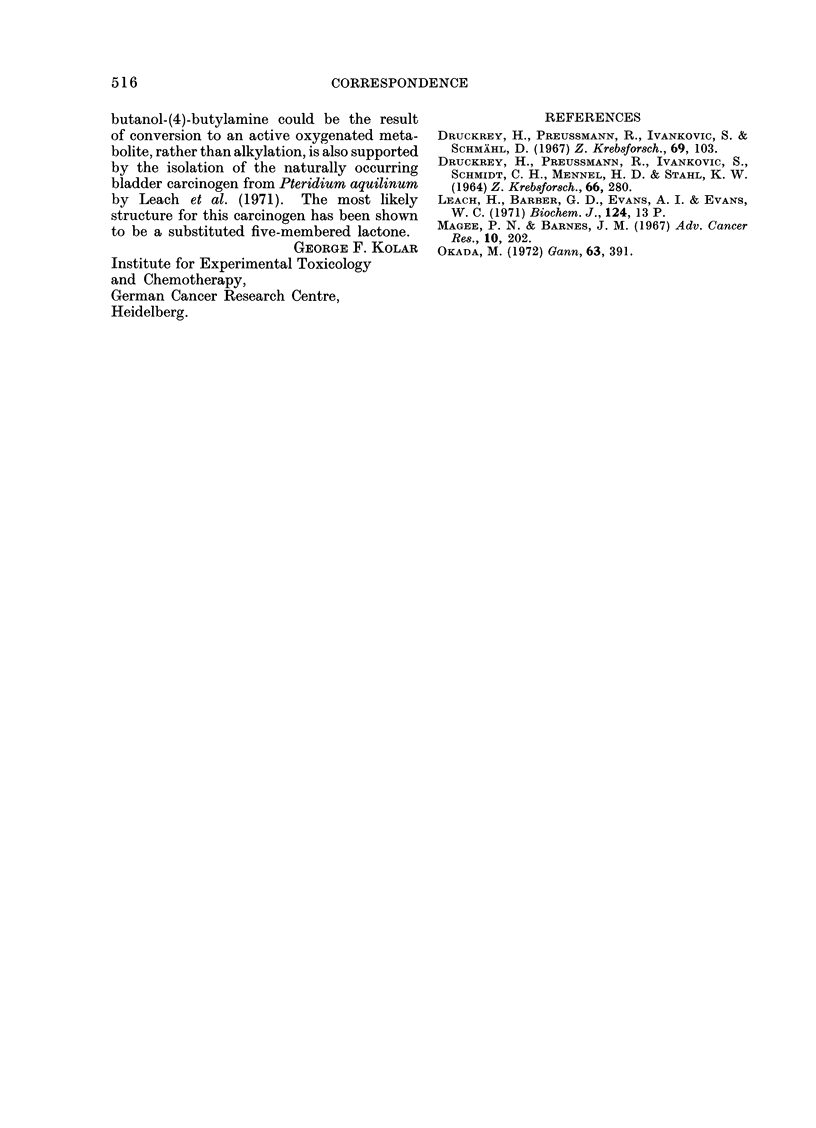# Induction of bladder cancer by N-nitroso-dibutylamine and N-nitrososo-butanol-(4)-butylamine.

**DOI:** 10.1038/bjc.1972.72

**Published:** 1972-12

**Authors:** G. F. Kolar


*Br. J. Cancer* (1972) **26**, 515.

### *Correspondence*

### Induction of Bladder Cancer by *N*-Nitroso-Dibutylamine and *N*-Nitrososo-Butanol-(4)-Butylamine

Sir,—In a series of 65 *N*-nitroso compounds that have been screened for carcinogenic activity in rats by Druckrey *et al.* (1967), *N*-nitroso-dibutylamine was found to be the only symmetrical dialkylnitrosamine which induced cancer of the urinary bladder. Similarly, the related *N*-nitroso-butanol-(4)- butylamine (I) was reported to be an even more potent and specific bladder carcinogen, since cancer of this organ was diagnosed in all 25 animals which had received daily doses of the compound as low as 20 mg/kg in drinking water (Druckrey *et al.*, 1964). The available evidence indicates that the nitrosamines are metabolized into potential alkyl donors and aldehydes (Magee and Barnes, 1967) and their carcinogenic activity has been attributed to alkylation of biopolymers. The alkylating intermediates have been thus regarded key metabolites in nitrosamine carcinogenesis, whereas the functional importance of the aldehydic fragments has been hitherto neglected.

Experimental bladder cancer was induced by nitrosamines substituted with n-C4 alkyl groups only and the activity towards bladder epithelium was greatly increased by the presence of an ω-hydroxyl group in one of the alkyl chains. It is therefore conceivable that a unique metabolite which could be derived from the structural elements of the applied nitrosamine, was responsible for the specific biological effect. On the assumption that enzymic dealkylation is also an essential step involved in the activation of *N*-nitroso-butanol-(4)-butylamine, the fission of the hydroxylated molecule would result in the liberation of 4-hydroxybutanal (II) which would readily cyclize to its tautomeric hemiacetal, 2-hydroxytetrahydrofuran (III). Subsequent oxidation of this intermediate to γ-butyrolactone can be easily envisaged. Alternatively, and even more likely, *in vivo* oxidation of the alcohol group in (I) to a carboxyl (IV), followed by enzymic hydroxylation and cleavage, would lead to the release of 4-butanal-1-carboxylic acid (V) which would also cyclize to its tautomeric lactol (VI). The mechanistic feasibility of the latter pathway is supported by a recent finding of Okada (1972) that *N*-nitroso-butanol-(4)-butylamine is metabolized in the rat to *N*-nitroso-butanoic acid-(4)-butylamine (IV) in 20% yield; this compound was also shown to be a powerful bladder carcinogen. The existence of similar ring-chain equilibria in biologically active molecules, such as penicillic acid, is a well-known phenomenon. The sequence of suggested transformations is shown below.

The proposed metabolic pathway would therefore establish a relationship between the nitrosamines and the lactones which are another class of known chemical carcinogens. Since further *in vivo* modifications of the applied nitrosamine can occur, the ultimate carcinogen could well be a related lactone derived from the modified C4 skeleton.

Despite much intensive research, the molecular basis of chemical carcinogenesis is still obscure, and any attempt to correlate chemical structure with carcinogenic activity is therefore a most difficult task. Nevertheless, the proposal that bladder carcinogenesis by *N*-nitroso-dibutylamine and *N*-nitroso-

Possible transformations of *N*-nitroso-butanol-4-butylamine *in vivo*.

butanol-(4)-butylamine could be the result of conversion to an active oxygenated metabolite, rather than alkylation, is also supported by the isolation of the naturally occurring bladder carcinogen from *Pteridium aquilinum* by Leach *et al.* (1971). The most likely structure for this carcinogen has been shown to be a substituted five-membered lactone.

GEORGE F. KOLAR
Institute for Experimental Toxicology and Chemotherapy,
German Cancer Research Centre,
Heidelberg.

REFERENCES

DRUCKREY, H., PREUSSMANN, R., IVANKOVIC, S. & SCHMÄHL, D. (1967) *Z. Krebsforsch.*, **69**, 103.
DRUCKREY, H., PREUSSMANN, R., IVANKOVIC, S., SCHMIDT, C. H., MENNEL, H. D. & STAHL, K. W. (1964) *Z. Krebsforsch.*, **66**, 280.
LEACH, H., BARBER, G. D., EVANS, A. I. & EVANS, W. C. (1971) *Biochem. J.*, **124**, 13 P.
MAGEE, P. N. & BARNES, J. M. (1967) *Adv. Cancer Res.*, **10**, 202.
OKADA, M. (1972) *Gann*, **63**, 391.